# Researching glutamate – induced cytotoxicity in different cell lines: a comparative/collective analysis/study

**DOI:** 10.3389/fncel.2015.00091

**Published:** 2015-03-17

**Authors:** Aristeidis A. Kritis, Eleni G. Stamoula, Krystallenia A. Paniskaki, Theofanis D. Vavilis

**Affiliations:** Laboratory of Physiology, Department of Physiology and Pharmacology, School of Medicine, Faculty of Health Sciences, Aristotle University of Thessaloniki, ThessalonikiGreece

**Keywords:** excitotoxicity, glutamate oxidative toxicity, PC12, SH-SY5Y, HT-22, NT-2, RGC-5, SCN2.2

## Abstract

Although glutamate is one of the most important excitatory neurotransmitters of the central nervous system, its excessive extracellular concentration leads to uncontrolled continuous depolarization of neurons, a toxic process called, excitotoxicity. In excitotoxicity glutamate triggers the rise of intracellular Ca^2+^ levels, followed by up regulation of nNOS, dysfunction of mitochondria, ROS production, ER stress, and release of lysosomal enzymes. Excessive calcium concentration is the key mediator of glutamate toxicity through over activation of ionotropic and metabotropic receptors. In addition, glutamate accumulation can also inhibit cystine (CySS) uptake by reversing the action of the CySS/glutamate antiporter. Reversal of the antiporter action reinforces the aforementioned events by depleting neurons of cysteine and eventually glutathione’s reducing potential. Various cell lines have been employed in the pursuit to understand the mechanism(s) by which excitotoxicity affects the cells leading them ultimately to their demise. In some cell lines glutamate toxicity is exerted mainly through over activation of NMDA, AMPA, or kainate receptors whereas in other cell lines lacking such receptors, the toxicity is due to glutamate induced oxidative stress. However, in the greatest majority of the cell lines ionotropic glutamate receptors are present, co-existing to CySS/glutamate antiporters and metabotropic glutamate receptors, supporting the assumption that excitotoxicity effect in these cells is accumulative. Different cell lines differ in their responses when exposed to glutamate. In this review article the responses of PC12, SH-SY5Y, HT-22, NT-2, OLCs, C6, primary rat cortical neurons, RGC-5, and SCN2.2 cell systems are systematically collected and analyzed.

## Introduction

Glutamate is one of the main excitatory neurotransmitters of the CNS, contributing to normal neural transmission, development, differentiation, and plasticity. However, excessive extracellular glutamate concentration can lead to uncontrolled continuous depolarization of neurons, a toxic process called, excitotoxicity, leading eventually to neuronal death. Excitotoxicity is associated with many neurodegenerative conditions such as Huntington’s disease, Alzheimer’s disease, lateral amyotrophic sclerosis, Parkinson’s disease and stroke or traumatic brain injury.

Glutamate both in neurons and glial cells is synthesized through the tricarboxylic acid cycle and additionally in neurons by the glutamate–glutamine cycle, where it is accumulated in vesicles for future release. Glutamate is ligand to post-synaptic either iGluRs or mGluRs. Under pathological stimuli, glutamate release is excessive; GluR over activation ensues, resulting in an augmented intracellular Ca^2+^ influx.

Increased intracellular Ca^2+^ concentration disrupts calcium homeostasis and initiates a cascade of signaling pathways, leading to up regulation of nNOS, dysfunction of mitochondria, deregulation of oxidative phosphorylation, ROS production, ER stress, and release of lysosomal enzymes. Excessive calcium concentration is the key mediator of glutamate toxicity through over activation of ionotropic and metabotropic receptors. In addition, glutamate accumulation can also inhibit CySS uptake by reversing the action of the CySS/glutamate antiporter (Xc^-^). Reversal of Xc^-^ action reinforces the aforementioned events by depleting neurons of CySS and eventually GSH, leading to free radical accumulation. In the absence of glutamate receptors, glutamate toxicity can occur through this antiporter promoting a Ca^2+^ independent, non-receptor mediated oxidative glutamate toxicity. Glutamate exerts its toxic effects through molecular pathways, which lead to neurodegeneration and cell death, for reviews see (Wang and Qin, 2010; Lai et al., 2014).

In the last three decades various cell models have been used in excitotoxicity studies and different pathways pertaining to cell survival and/or cell death have been reported to be triggered in each cell line. This review summarizes the effect of excitotoxicity on the homeostasis of the cellular organelles, the cell signaling pertaining to survival and cell death and focuses on the cell lines that have been used as models for the study of the excitotoxicity.

## Glutamate-Induced Cytotoxicity Triggering and the Effect on Intracellular Organelles

### Glutamate Release and Reuptake: the Glutamate–Glutamine Cycle and Xc^-^

During neurotransmission, glutamate is released by depolarization of pre-synaptic membranes via a Ca^2+^-dependent process, involving VDCCs (Meldrum, 1994; Anderson and Swanson, 2000). VDCCs are of N, P/Q, R, and L-type characterized by their subunit composition and their inhibition by specific toxins. They mediate glutamate synaptic release in CNS and their distribution among nerve terminals varies. In certain terminals, only one type is present, while others possess more than one (Reid et al., 2003).

**FIGURE 1:**
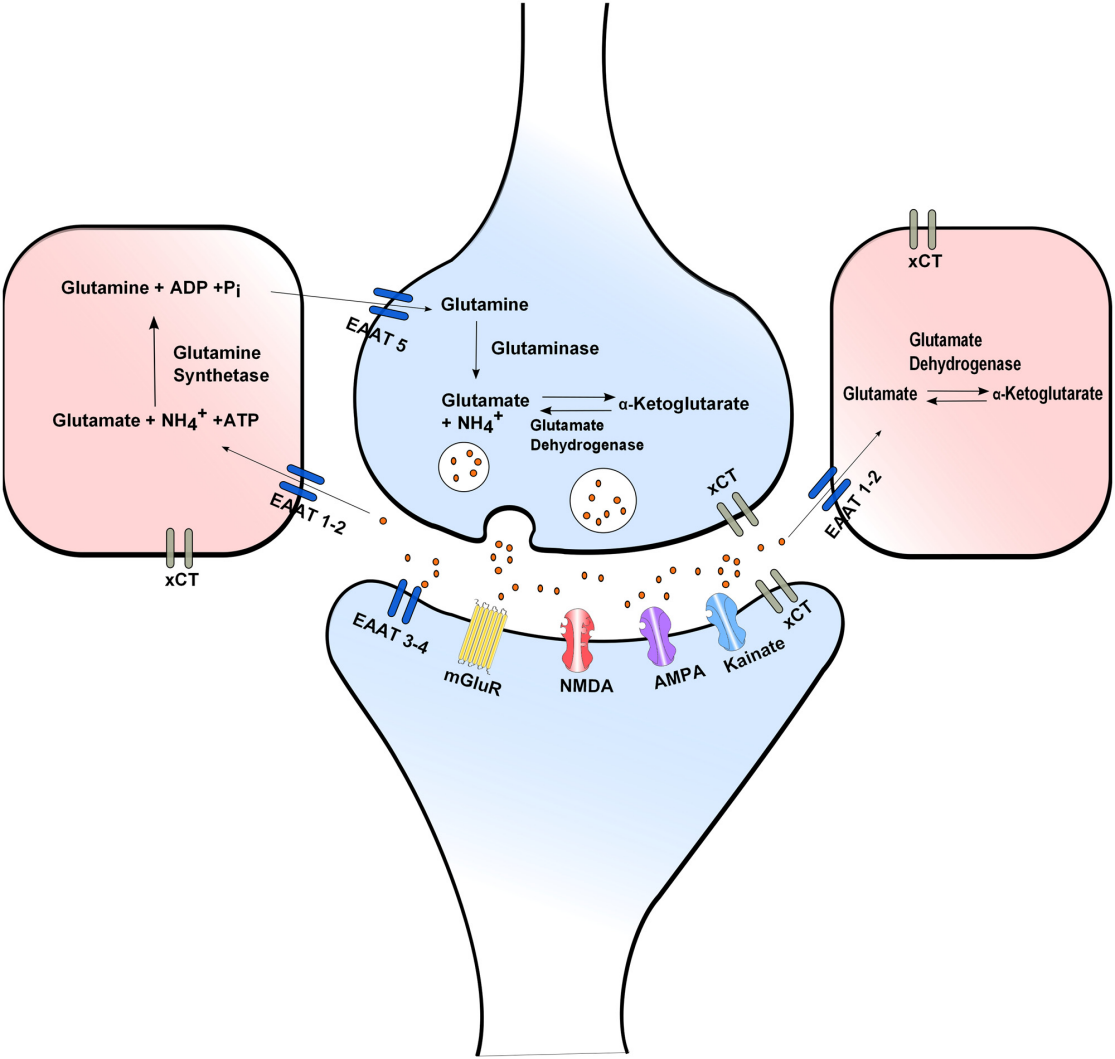
**Glutamate release and uptake, the Xc^-^ antiporter and the glutamate/glutamine cycle.** In glial cells reuptaken glutamate is converted to glutamine by glutamine synthetase. Glial glutamine is taken up into the presynaptic neuron via Na^+^-dependent glutamine uptake systems, where it is converted to glutamate by glutaminase. Extra- and intra-cellular glutamate concentrations are modulated through the X**_C_**^-^ antiporter. Neurotransmission is ended by efficient glutamate reuptake via Na^+^-dependent high affinity glutamate membrane EAATs.

Neurotransmission is ended within millisecond by efficient glutamate reuptake via Na^+^-dependent high affinity glutamate membrane EAATs: EAAT1 (GLAST), EAAT2 (GLT1), EAAT3 (EAAC1), EAAT4, and EAAT5. EAAT2 is commonly expressed in glial cells and EAAT3 in neurons. EAAT2 is believed to play the main role in regulating extracellular glutamate concentration (Danbolt, 2001). In glial cells reuptaken glutamate is converted to glutamine by glutamine synthetase thus ending neurotransmission, offering neuroprotection and preventing excitotoxicity. Glial glutamine is taken up into the presynaptic neuron via Na^+^-dependent glutamine uptake systems, where it is converted to glutamate by glutaminase thus completing the glutamate–glutamine cycle (Attwell, 2000; Daikhin and Yudkoff, 2000; Hertz et al., 2007; **Figure 1**).

The Ca^2+^-independent glutamate release is attributed to reverse action of the aforementioned glutamate transporters. Reverse action of glutamate transporters can occur during depolarization, when the Na^+^ and K^+^ gradients are diminished possibly contributing to the regulation of glutamatergic neurotransmission. In hypoxia reduced expression of EAAT1 and 2 contributes to increase extracellular glutamate concentration leading to neuronal overexcitation and excitotoxicity (Rossi et al., 2007; Niciu et al., 2012).

Another important molecule modulating both extra- and intra-cellular glutamate concentrations is the X**_C_**^-^. This is of added importance for the nervous system since L-cystine taken up by the cells can be used for GSH synthesis and protection from oxidative insults, for a review see (Bridges et al., 2012). Excessive extracellular glutamate concentration blocks the uptake of CySS which is essential for biosynthesis of GSH. GSH depletion influences the capacity of cells to scavenge free radicals, a fact that makes cells vulnerable to secondary events such as accumulation of ROS and alteration in Ca^2+^ homeostatic mechanisms resulting in cell death (Fukui et al., 2009).

### Glutamatergic Neurotransmition: the Glutamate Receptors

There are two types of glutamate receptors categorized according to their function. iCluRs functioning upon binding of glutamate as ion channels and mGluRs. mGluRs are G protein coupled receptors, coupled to their associated ion channels via a second messenger cascade. iGluRs are named after their respective agonist, NMDARs, AMPARs, and KARs. They are multimeric assemblies of different protein subunits that form homo or heteromeric complexes of varying subunit combination, resulting to multiple types of ion channels with different properties. iGluRs mediate fast synaptic transmission and are broadly classified in two classes as NMDA and non-NMDA receptors (Keinanen et al., 1990; Monyer et al., 1992; Nakanishi et al., 1994).

**FIGURE 2:**
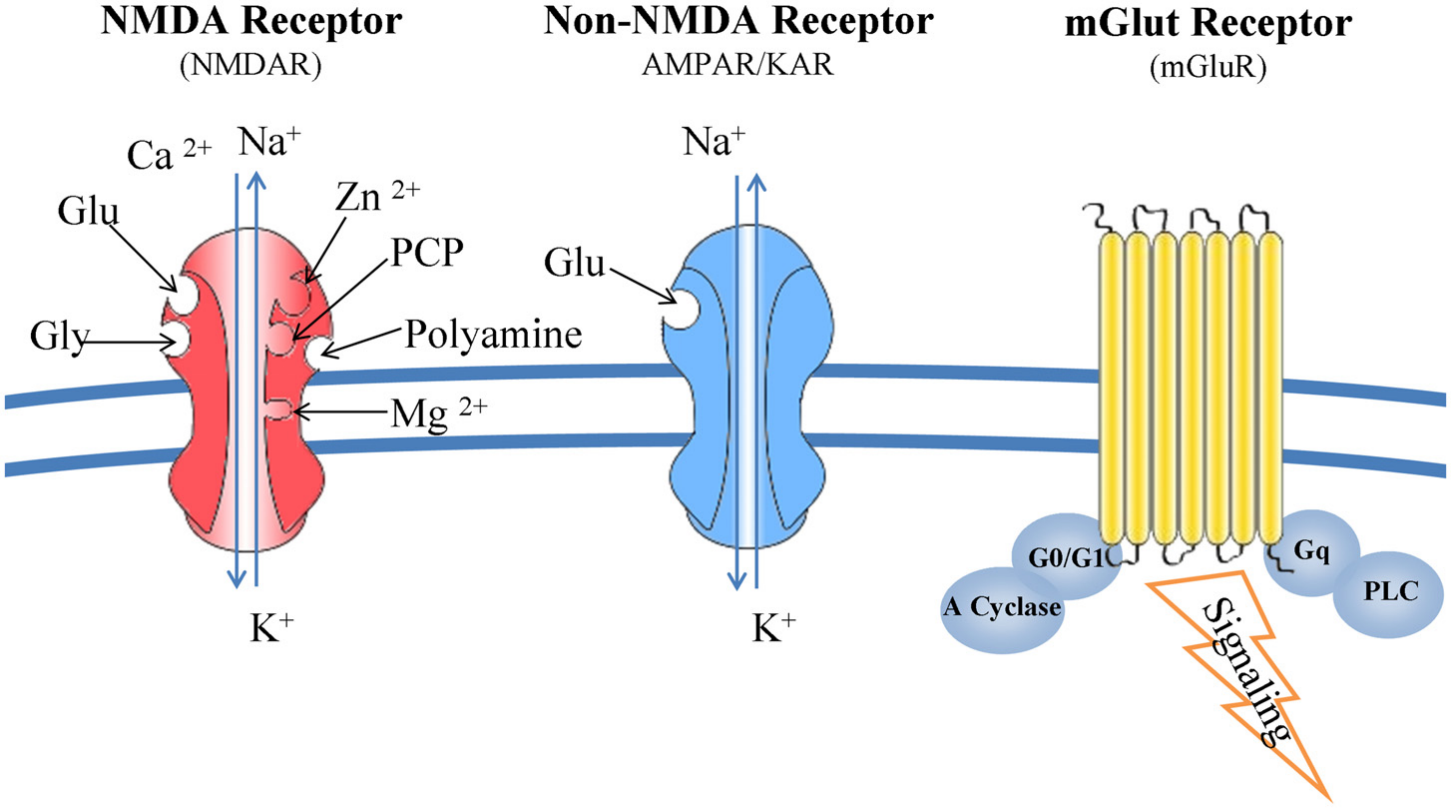
**Glutamate receptors: structure and function.** NMDARs bind glutamate, glycine, Mg^2+^, Zn^2+^, and polyamines. Composed from seven subunits (one NR1, four NR2, and two NR3), their function is determined by the combination of NR1 and NR2 subunits. NMDARs form channels that are more permeable to Ca^2+^ than Na^+^ and K^+^. Kainate and AMPA receptors interact only with glutamate and their specific agonists, and their associated channels are more permeable to Na^+^ and K^+^ than Ca^2+^. mGluRs are G-protein coupled receptors and trigger a second messenger cascade. They are found both at the pre- and post-synaptic neurons, subunits of metabotropic receptors are also expressed in microglia.

*N*-methyl-D-aspartate receptors are complex structures able to bind glutamate, glycine, Mg^2+^, Zn^2+^, and polyamines. Composed from seven subunits (one NR1, four NR2, and two NR3), their function is determined by the combination of NR1 and NR2 subunits. NMDARs form channels that are more permeable to Ca^2+^ than Na^+^ and K^+^. Upon binding of glutamate the magnesium ions, blocking the ion channel, are released and consequently the ion channel is activated allowing the influx of the aforementioned ions into the cytoplasm (Mehta et al., 2012). Kainate and AMPA receptors interact only with glutamate and their specific agonists, and their associated channels are more permeable to Na^+^ and K^+^ than Ca^2+^(Kostandy, 2012; **Figure 2**).

Metabotropic glutamate receptors are G-protein coupled receptors and trigger a second messenger cascade (Skeberdis et al., 2001a; Lea et al., 2005). They are found both at the pre- and post-synaptic neurons, but also subunits of metabotropic receptors are expressed in microglia (Janssens and Lesage, 2001; Byrnes et al., 2009; **Figure 2**).

There are eight different types of mGluRs (mGluR_1_ to mGLUR_8_) and are classified to three groups according to their structure and physiological activity (groups I, II, and III). Group I (mGluR 1 and 5) are mainly post-synaptic receptors coupled to a G_q_ heterotrimeric G protein. Upon binding of glutamate, PLC is activated and 1,4,5-inositol triphosphate is produced initiating multiple intracellular responses. Evidence is presented for PKC-dependent and independent pathways potentiating the NMDA responses by mGluRs (Kelso et al., 1992; Ugolini et al., 1997; Skeberdis et al., 2001b). Groups II and III are coupled to G_i_/G_0_ thus inhibiting the action of adenylate cycle and reducing intracellular cAMP levels.

Pertaining to excitotoxicity, NMDARs play the most important role as larger quantities of Ca^2+^ ions can be moved through them (Bloom, 1994). The role of kainate and AMPA receptors is also important as it has been shown that their activation is directly related to the ER stress. Activation of the iGluRs creates a depolarization (excitatory post-synaptic current) and depending on the number of the receptors that are activated, this current, can lead to an action potential. This current is also essential to the function of NMDARs since it can lift their voltage dependent Mg^2+^ block (Johnson and Ascher, 1990).

Of the mGluRs class I is positively connected with excitotoxicity by modulating the NMDA receptor activity while classes II and III are negatively related to the phenomenon, through the suppression of intracellular cAMP levels inhibiting the export of potentially neurotoxic glutamate from microglia offering a neuroprotective role (Ambrosini et al., 1995; Faden et al., 1997; Allen et al., 1999; McMullan et al., 2012).

### Glutamate Receptors and Cell Death

Both iGluRs and mGluRs mediate the excitotoxic insult via distinct, but not independent pathways. However, key players in the initiation of the exitoxic insult are the iCluRs. The elevated Ca^2+^ concentration upregulates the activity of nNOS, contributes to mitochondrial activity deregulation and ER stress, leading to cell membrane depolarization and dysfunction of intracellular organelles. As iGluRs are permeable not only to Ca^2+^ but also to Na^+^ and K^+^, their activation leads to depolarization of the cell membrane and osmotic inflow of water.

*N*-methyl-D-aspartate receptor activity can be modulated by mGluRs. mGluRs do not participate directly in the excitotoxic insult, but rather seem to modulate it. NMDARs can be up regulated by Src kinase mediated tyrosine phosphorylation, leading to increased channel permeability (Salter, 1998; Ali and Salter, 2001). Group I are positively coupled via a G_q_-protein to PLC, resulting in the release of Ca^2+^ from intracellular stores (Skeberdis et al., 2001a). Augmented intracellular Ca^2+^ concentration can lead to the sequential activation of PKC, Pyk2 and Src, resulting in the tyrosine phosphorylation of NMDARs (Lu et al., 2011). NMDAR phosphorylation via Src family results in its up regulation (Dikic et al., 1996; Salter, 1998) and increases open channel probability (Salter, 1998; Ali and Salter, 2001). PKC and mGluR 1 potentiate NMDARs currents not only by increased open channel probability but also by recruiting new channels to the membrane in cooperation with cytoskeletal proteins (Lan et al., 2001).

**FIGURE 3:**
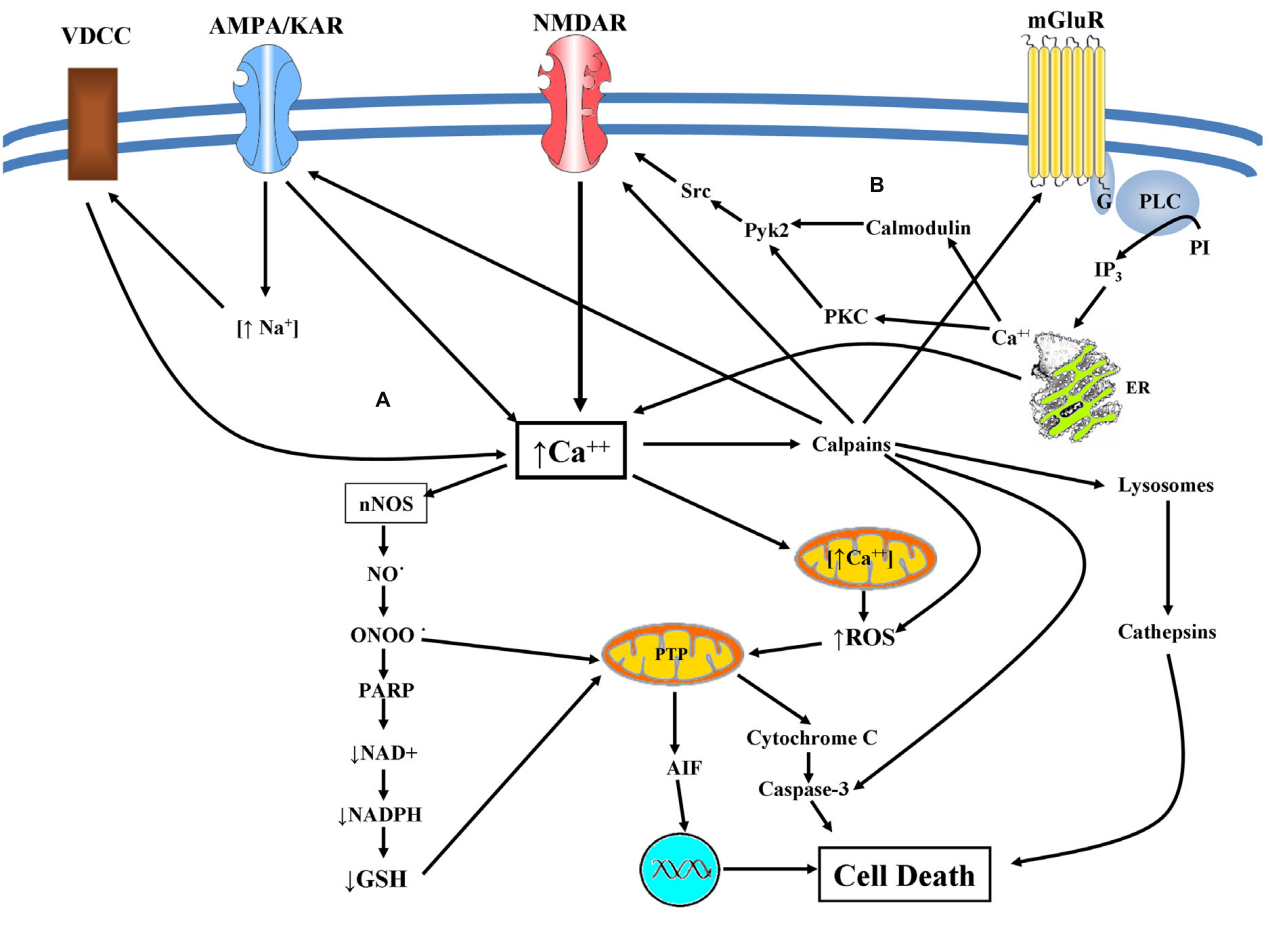
**(A)** Excitotoxicity mediated cell death: glutamate excitotoxicity causes Ca^2+^ mediated NO production leading to mitochondrial dysfunction resulting in superoxide production. Peroxynitrite is produced causing lipid peroxidation, direct DNA damage, and protein dysfunction. Peroxynitrite inhibits the mitochondrial electron transport chain, cytochrome c normal activity as well as of superoxide dismutase via protein nitration. Activation of ryanodine receptors in conjunction with accumulation of misfolded proteins and depletion of endoplasmic Ca^2+^ storage, results in ER dysfunction (ER-stress). These facts can provoke caspase mediated cell death and an eventual apoptotic cell death. Alternatively augmented intracellular Ca^2+^ concentration can lead to calpain activation engaging both calpain dependent and cathepsin dependent cell death. **(B)** mGluRs and NMDAR crosstalk: both mGluR1 and mGluR5 lead to the potentiation of NMDA receptor currents via Src-dependent mechanism in a PKC or calmodulin depended manner.

Both mGluR1 and mGluR5 lead to the potentiation of NMDA receptor currents via Src-dependent mechanism. Alternatively others (Minakami et al., 1997; Ishikawa et al., 1999; Shinohara et al., 2001) support that both mGluR1 and mGluR5 can interact with calmodulin in a calcium dependent manner, proposing that other molecules besides PKC may be responsible for the mGluR1 mediated activation of Pyk2/Src and the potentiation NMDARs currents (**Figure 3**). Group II (mGluR2 and 3) and III (mGluR4, 6, 7, and 8) are coupled to G_i_/G_0_ proteins leading to inhibition of adenylate cyclase decreasing the levels of cAMP in the cytoplasm (Conn and Pin, 1997). Subunits of metabotropic receptors (groups I, II, and III) are also expressed in microglia. Groups II and III through the suppression of intracellular cAMP levels inhibit the export of potential neurotoxic glutamate from microglia offering a neuroprotective role (McMullan et al., 2012).

Recently it is reported that calpains, proteases activated by elevated intracellular calcium concentration, form a negative feedback loop for controlling calcium influx, by means of cleavage and subsequent inactivation of glutamate receptors. This feedback loop may contribute to a neuroprotective effect against the excitotoxic insult (Doshi and Lynch, 2009; McMullan et al., 2012; **Figure 3**).

### nNOS Up Regulation and Cell Death the Key Player

*N*-methyl-D-aspartate receptors are linked to neuronal nitric oxide synthase (nNOS), and therefore their activation leads to production of toxic NO-levels. NMDARs and nNOS are connected by the post-synaptic density protein PSD-95. Over activation of NMDARs under excitotoxic conditions provokes Ca^2+^ influx which results in activation of nNOS via calmodulin (Schrammel et al., 2003; Brown, 2010).

More recently, NO has been shown to interfere with neurotoxicity through an interaction with GAPDH (Hara et al., 2005). GAPDH/Siah 1(an E3 ubiquitin ligase) pathway is a novel pathway that links NO to a form of apoptotic-like death (Leach et al., 1986; Hara et al., 2005). Increased intracellular concentration of NO causes *S*-nitrosylation of GAPDH which triggers GAPDH binding to Siah 1. GAPDH/Siah1 complex is translocated to the nucleus resulting in the transcription of the proapoptotic factor p53 (Sen et al., 2008). Activation of nuclear proteins such as p53 results in pyknotic nucleus and other morphological characteristics that indicate apoptotic death (Sen et al., 2009). Moreover, GAPDH plays a pivotal role in glycolysis and it is possible that GAPDH-NO interaction leads to loss of function and energy failure (Vedia et al., 1992).

Glutamate excitotoxicity causes Ca^2+^ mediated NO production leading to mitochondrial dysfunction resulting in superoxide production, which is released via voltage dependent anion channels into the cytosol. NO-superoxide interaction can produce peroxynitrite an oxidative molecule that can cause lipid peroxidation, direct DNA damage and protein dysfunction (Radi et al., 1991a,b; Salgo et al., 1995). Peroxynitrite has many deleterious effects, such as the inhibition of mitochondrial electron transport chain (Radi et al., 1994), cytochrome c normal activity (Nakagawa et al., 2001) as well as of superoxide dismutase via protein nitration (Yamakura et al., 1998). These facts can provoke caspase mediated cell death and an eventual apoptotic cell death (Szab, 2003; **Figure 3**).

In addition NO activated soluble guanylate cyclase increase levels of cGMP. The three principal targets of cGMP are protein kinase G (PKG), cyclic-nucleotide gated channels (CNGCs), and cyclic-nucleotide phosphodiesterase (PDE). Of these, PKG provides the broadest means for controlling ion-channel function modulating this way neuronal excitability (Denninger and Marletta, 1999; Ahern et al., 2002).

### Deregulation of Intracellular Organelles-Mitochondria, ER, Lysosomes

Deregulation of cell signaling due to excitotoxicity leads to altered function of intracellular organelles. Mitochondria, ER, and lysosomes are mostly affected by the increased Ca^2+^ influx, and their degeneration plays a pivotal role in neuronal death.

*Mitochondria* produce not only ATP, but also ROS and regulate Ca^2+^ homeostasis. Normally Ca^2+^ intake controls the activity of three dehydrogenases: pyruvate, isocitrate and ketoglutaric acid dehydrogenase, as well as ATP synthase. However, the increased influx of Ca^2+^ leads to mitochondrial Ca^2+^ overload and depolarization of mitochondrial membrane. The consequences of this overload is: (a) the activation of mitochondrial permeability transition pore, (b) phospholipase A2 and xanthine oxidase up-regulation, (c) inhibition of respiratory chain enzymes and (d) deactivation of catalase, superoxide dismutase, and GSH peroxidase (Yang et al., 2011; Cheng et al., 2012). The deregulation of respiratory chain enzymes firstly decreases ATP synthesis and secondly overproduces ROS, which cannot be neutralized by the cell. ATP depletion leads to neuronal bioenergetic failure and neurodegeneration. ROS react with biological molecules (lipids, proteins, nucleic acids, carbohydrates), producing new oxidative species, which trigger oxidative chain reactions of other macromolecules. In this way ROS bind to DNA evoking its fragmentation. Mitochondrial DNA, which lacks on histones, is especially vulnerable to ROS oxidation. The above in combination to PLC up-regulation, lead to membrane lipid peroxidation with the consequent membrane destabilization (Nicholls and Budd, 1998).

The result of all these factors is synaptic dysfunction, impaired neuronal plasticity and cell death via apoptosis, necrosis and/or autophagy. A central player in the potential driven mitochondrial Ca^2+^ uptake, is the mitochondrial Ca^2+^ uniporter (MCU), whose gene has been recently characterized (Luetjens et al., 2000; Pivovarova et al., 2004). In excitotoxicity Ca^2+^ uniporter acts as a mediator of death-signal, induced by loss of mitochondrial membrane potential (MMP), but can also serve a pro-survival role through neuroprotective Ca^2+^ signaling stemming from synaptic activity (Qiu et al., 2013; **Figure 3**).

*Endoplasmic reticulum* is an important cell organelle responsible for correct folding and sorting, translation, and post-translational modification of proteins and serves as an intracellular Ca^2+^ storage. ER is functionally connected to mitochondria through intracellular Ca^2+^ flow between them. ER functions can be disturbed by different insults such as accumulation of unfolded proteins and changes in Ca^2+^ homeostasis. Overstimulation of AMPA receptors results in inordinate Ca^2+^ concentration which leads to activation of ryanodine receptors (RyRs) located in ER (Ruiz et al., 2010; Mehta et al., 2012).

Activation of RyRs in conjunction with accumulation of misfolded proteins and depletion of endoplasmic Ca^2+^ storage, results in ER dysfunction (ER-stress). Cell response to ER-stress is called unfolded protein response (UPR; Boyce and Yuan, 2006) and consists of two repair mechanisms: activation of proteasome and ubiquitinization of dysfunctional proteins and induced expression of molecular chaperones (Verkhratsky, 2005; Boyce and Yuan, 2006; Ruiz et al., 2010).

*Lysosomes* are organelles which contain hydrolytic enzymes (proteases, nucleases, and lipases) necessary for intracellular digestion. Under excitotoxic conditions the number of lysosomes is increased because of enhanced induction of autophagy. It has been reported that NMDARs channeling in rat cerebellar granule neurons in culture, increased phaghosomes and their conjugation with lysosomes (Sadasivan et al., 2010). Moreover, several lines of evidence support a cross-talk between apoptosis and autophagy, since certain caspases can directly or indirectly activate cathepsins (Hsieh et al., 2009). Mitochondrial dysfunction leads eventually to activation of caspases which results in the release of cathepsins. The latter activates authophagy through release of lysosomal contents into the cytoplasm (Nixon et al., 2001; Terman et al., 2006).

Autophagy is a natural cell function in CNS since it plays a pivotal role in neuroprotection. It has been reported that traumatic brain injury is followed by enhanced autophagic processes. Activation of NR2B via CaMKII kinases contributes to the release of Beclin-1 which induces autophagy, while NR2B antagonists prevent excitotoxic-induced autophagy. Over activation of lysosomes and uncontrolled release of lysosomal enzymes caused by excitotoxicity contributes to neuronal death and brain pathology (Sadasivan et al., 2010; **Figure 3**).

## Cell Lines Models Utilized for the Study of Glutamate-Induced Cytotoxicity

Based in bibliographic search we concluded that nine cell lines are widely used in researching *in vitro* excitotoxicity: PC12-rat pheochromocytoma, SH-SY5Y-human neuroblastoma, HT-22-immortalized mouse hippocampal cell line, Ntera /D1-NT-2 human teratocarcinoma, oligodendroglial lineage cells (OLCs), C6 -rat glioma, primary cortical rat neurons (PCRNs), RGC-5-mouse retinal ganglion cells, and SCN2.2 -hypothalamic suprachiasmatic nucleus (SCN) rat cell line. Below we summarize the methodology employed and the findings with respect to the signaling pathways activated in each cell line while researching excitotoxicity.

### PC12

PC12 is a cell line derived from rat adrenal medulla pheochromocytoma, they synthesize dopamine and glutamate and can be induced to differentiate by NGF to a sympathetic phenotype expressing neurites and excitability (Greene and Tischler, 1976). PC12 cell line has been extensively used as a tool for studying the function of neurons, neuronal differentiation, and neurotoxicity.

Glutamate exerts its toxic effects on PC12 in a dose and time dependent manner. Its toxic concentration varies between 0.01 and 10 mM (Pereira and Oliveira, 1997, 2000; Penugonda et al., 2006; Pourzitaki et al., 2007, 2008, 2009; Lu et al., 2011) while time varies from 30 min to 3-12-24-48 h of incubation.

Even though mRNA for NMDA receptor subunits is expressed by PC12 cells (Schubert et al., 1992; Leclerc et al., 1995) there are contradictory findings, reported in the literature, concerning the receptor functionality, and presence of receptor protein. According to Sucher et al. (1993) only trace amounts of the receptor protein are present in PC12 cells and no functional NMDA-operated channels exist in this cells line. This has also been supported recently from *in vitro* findings in PC12 cell studies (Said et al., 1998; Vazhappilly and Sucher, 2002; Edwards et al., 2007). On the other hand, others support both the presence and functionality of the NMDARs in PC12 cells (Casado et al., 1996; Penugonda et al., 2006; Pourzitaki et al., 2007, 2008, 2009; **Figure 3**).

**FIGURE 4:**
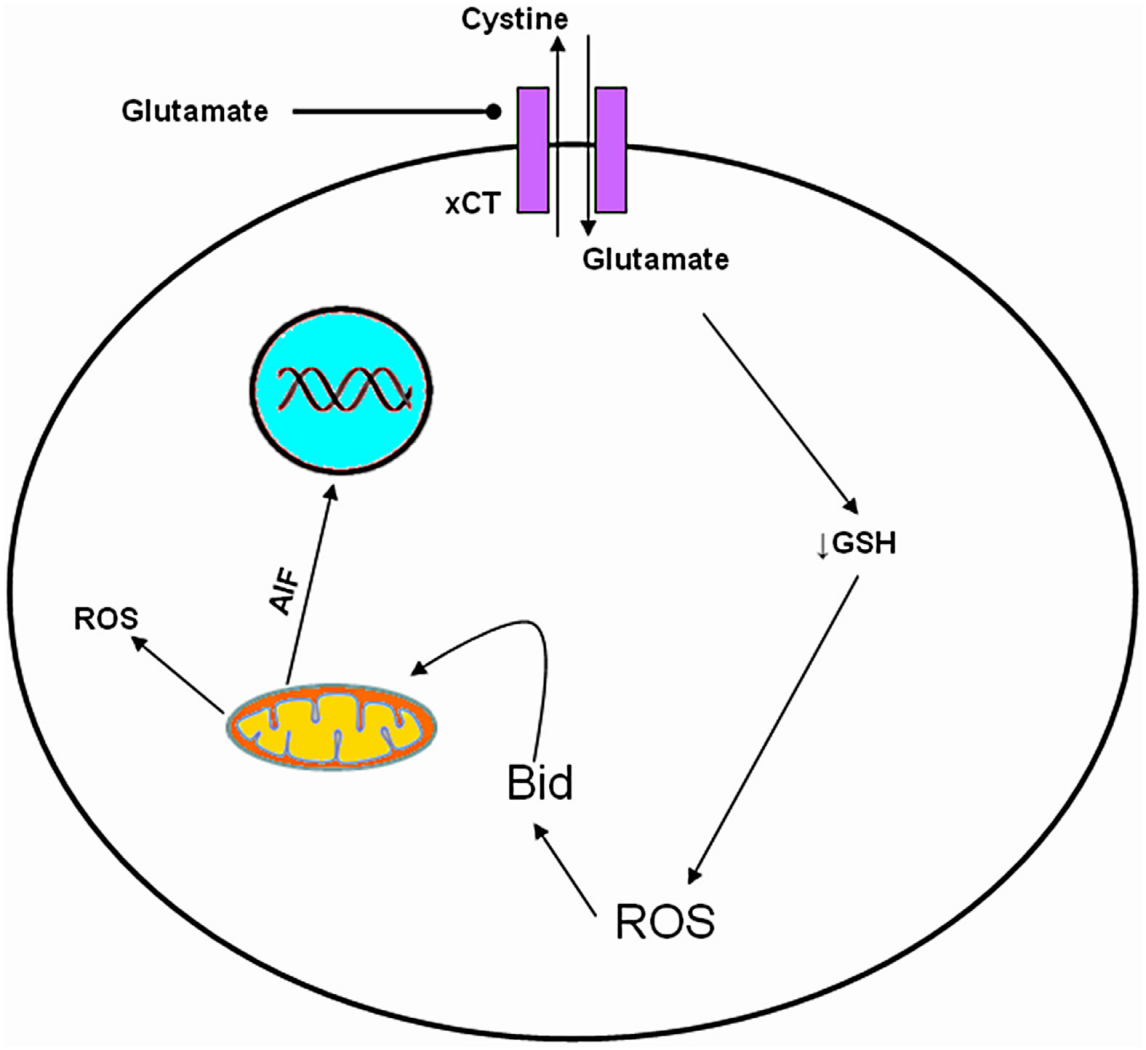
**Molecular mechanism of oxidative glutamate toxicity.** Increased extracellular glutamate concentration leads to reverse action of Cys glutamate antiporter. GSH depletion, follows due to decrease of intracellular Cys influencing the capacity of cells to scavenge free radicals, rendering them vulnerable mitochondrial dysfunction and to secondary events such as accumulation of ROS, production of Bid and AIF, resulting in cell death.

In addition to excitotoxicity, glutamate appears to exert a cytotoxic action at very high extracellular concentrations (5–10 mM; Murphy et al., 1990). This glutamate-induced cytotoxicity is independent of NMDARs and is mediated through the inhibition of CySS uptake leading to depletion of GSH and oxidative glutamate toxicity (Tyurin et al., 1998; Penugonda et al., 2006; **Figure 4** and **Table 1**).

**Table 1:** Reported responses of individual cell lines to excitotoxicity and oxidative glutamate toxicity.

Cell lines	Excitotoxicity	Oxidative glutamate toxicity	Glutamate dose range	Time range of glutamate incubation
PC12	+	+	10 μM–10 mM	0.5–72 h^1^
SH-SY5Y		+	8–80 mM	0.5–48 h^2^
HT22		+	4–8 mM	3–48 h^3^
NT-2	+	+	10 μM–10 mM	4–58 h^4^
OLCs	+		50 μM–2 mM	12–24 h^5^
C6	+	+	10 μM–10 mM	1–28 h^6^
Primary astrocytes		+	2–5 mM	24–72 h^7^
PCRNs	+	+	1–5 mM	6–15 h^8^
RGC-5	+	+	1–25 mM	24–48 h^9^
SCN2.2	+		10 mM	>48 h^10^
GT1-7	+		10 mM	>48 h^10^

(+) positive for trait. ^1^(Casado et al., 1996; Pereira and Oliveira, 1997, 2000; Penugonda et al., 2006; Pourzitaki et al., 2007, 2008, 2009; Lu et al., 2011); ^2^(Yoshioka et al., 2000; Sun et al., 2010; Hu et al., 2012; Park et al., 2012; Nampoothiri et al., 2014); ^3^(Davis and Maher, 1994; Stanciu et al., 2000; Gursoy et al., 2001; Zhang and Bhavnani, 2006; Xu et al., 2007; Landshamer et al., 2008; Tobaben et al., 2011; Kumari et al., 2012); ^4^(Munir et al., 1995; Sandhu et al., 2003); ^5^(Oka et al., 1993; Yoshioka et al., 2000); ^6^(Han et al., 1997; Sribnick et al., 2006; Castillo et al., 2010a); ^7^(Oka et al., 1993; Obara-Michlewska et al., 2014); ^8^(Stanciu et al., 2000; Zhang and Bhavnani, 2006); ^9^(Aoun et al., 2003; Fan et al., 2006; Van Bergen et al., 2009); ^10^(Karmarkar et al., 2011; Karmarkar and Tischkau, 2013).

The above two mechanisms of glutamate-mediated toxicity can act cooperatively on PC12 cell death (Ma et al., 2012), while other investigators support that cell death on PC12 is exclusively due to NMDARs over activation (Penugonda et al., 2006; Pourzitaki et al., 2007, 2008, 2009) or inhibition of glu/cys antiporter without NMDAR implication (Pereira and Oliveira, 1997, 2000). We propose that these conflicting reports can be attributed to researchers studying cell death triggered simultaneously by two different events: increased intracellular Ca^2+^ concentration and GSH depletion. At glutamate concentrations greater than 20 μM the X_c_^-^ antiporter begins the reverse CySS transport thus depriving the cells of their ROS inactivation potential (Seib et al., 2011).

In relation to the above, there are reports that excitotoxic cell death in PC12 cells, can be effected by apoptosis and/or necrosis (Bal-Price and Brown, 2000; Lu et al., 2011; Ma et al., 2012), while others support a caspase independent calpain mediated cell death (Roth et al., 2000; Pourzitaki et al., 2007, 2009) probably necroptosis by activation of AIF (Shang et al., 2014). Investigating hypoxia in a PC12 oxygen glucose deprivation model (Kritis et al., 2011), showed that cathepsin D inhibition protects from cell death suggesting the implication of the autophagic processes. This is important in view of excitotoxicity following ischemic insults due to excessive cell death and uncontrolled release of glutamate (Lai et al., 2014).

Neuroprotective mechanisms of PC12 cells include the expression of GRP78, which suppresses oxidative stress and stabilizes Ca^2+^ homeostasis (Yu et al., 1999). Protein kinase B/Akt exhibits prosurvival and antiapoptotic activities and is involved in growth factor-mediated neuronal protection. Akt deactivation characterizes both caspase-dependent and -independent cell death (Luo et al., 2003). Additionally PI3K/Akt pathway may preferentially regulate both NGF and BDNF-mediated cell survival (Nguyen et al., 2010; **Table 2**).

**Table 2:** Reported effectors and molecular pathways in glutamate cytotoxicity.

Effectors-pathways	PC12	SH-SY5Y	HT-22	NT-2	OLCs	C6	PCRNs	RGC-5	SCN2.2	GT1-7
Ionotropic (iGluR)			-	+/+		+				+
NMDA	+/-	+/-						+	+	
AMPA/Kainate					+					
Metabotropic (mGluR)	+	+	-		+					+
Cystine/glutamate antiport	+	+	+	+			+			
GSH	↓	↓	↓	↓	↓	↓				
R.O.S	↑	↑	↑	↑	↑	↑				
Caspases	+	+	-	+	+			+	-	↑
Cytochrome c	+		+		+					
ERK pathway	+		+			+		+	+	↓
PkB/Akt/PI3 pathway	+		-					+	↑	
Rac-NADPH-ROS pathway(NF-κB)		+								
P38/MAPK pathway								↓	-	↑
JNK pathway			↑					↓	+	-
BDNF								+		
AIF and calpains	+		+		+					

*↑/↓: Increase/decrease respectively of the responding effectors or pathways under excitotoxicity effect. +: Presence of the responding effectors or pathways in the specific cell line. -: Absence of the responding effectors or pathways in the specific cell line. +/-: Both presence and absence of effectors or pathways reported for the same cell line.*

### Human Neuroblastoma SH-SY5Y Cell Line

Human brain neuroblastoma SH-SY5Y differentiated cell line is derived from bone marrow and is extensively used as a model for the study of oxidative stress pertaining to neuronal death. SH-SY5Y cells exhibit many characteristics of dopaminergic neurons, they have the ability to synthesize dopamine and norepinephrine and they express dopamine transporter. Upon treatment with a variety of agents, including RA (Singh and Kaur, 2009) phorbol ester 12-*O*-tetradecanoylphorbol-13-acetate (TPA; Pahlman et al., 1981), BDNF (Cernaianu et al., 2008), dibutyryl cAMP (Kume et al., 2008), purine or staurosporine (Mollereau et al., 2007). SH-SY5Y cells are differentiated and exhibit neuronal like characteristics (Cheng et al., 1995; Ismail et al., 2012). This cell line serves as a neuronal model for Parkinson’s disease research (Xie et al., 2010).

Several studies in this cell line have detected the expression of both iCluRs and mGluRs (Naarala et al., 1993; Nair et al., 1996; Sun and Murali, 1998; Naarala et al., 2002; Akundi et al., 2003), while others support that SH-SY5Y cells are deficient in NMDARs or that NMDARs have no function (Cheng et al., 1995; Sun et al., 2010; Xie et al., 2010; Ismail et al., 2012). Sun et al. (2010) support that increased cytoplasmic Ca^2+^ after glutamate treatment is independent of glutamate receptors (both NMDA and metabotropic) in SH-SY5Y cells. In this cell line glutamate induced cytotoxicity could be mediated by oxidative stress through CySS/glutamate antiporter, depletion of GSH, down regulation of SOD activity leading to apoptosis (oxidative glutamate toxicity; **Figure 4** and **Table 1**).

Others (Fallarini et al., 2009; Beske and Jackson, 2012) use the SH-SY5Y cell line for investigating hypoxia employing protocol of oxygen glucose deprivation (OGD). Experimental evidence suggests that glutamate-induced apoptotic cell death involves the Rac-NADPH oxidase-mediated ROS formation in SH-SY5Y cells (Nikolova et al., 2005; **Table 2**). One of the downstream targets of NADPH oxidase-derived superoxide radicals is the transcription factor NF-κB which regulates the expression of many genes involved in cell survival and inflammation. NF-κB is also a key factor in regulating NADPH oxidase expression and it is possible that there is a positive feedback loop in which NF-κB activation by oxidative stress leads to further radical production via NADPH oxidase (Nikolova et al., 2005; Anrather et al., 2006).

### HT-22 Immortalized Hippocampal Cell Line

HT-22 cell line is an immortalized mouse hippocampal cell line that is extensively used to study the non-receptor mediated oxidative glutamate toxicity (Murphy et al., 1989, 1990; Davis and Maher, 1994). These cells lack iCluRs but are still sensitive to high concentrations of extracellular glutamate (**Tables 1** and **2**). Glutamate evokes oxidative death in HT22 in a time- and dose-dependent manner involving both necrotic and apoptotic processes (Tan et al., 2001; Fukui et al., 2009; Xu et al., 2010). Recent data shows that glutamate induces mainly necrosis at early time points (before 12 h), but predominantly induces apoptosis at latter ones (12–24 h; Thomas and Huganir, 2004; Fukui et al., 2009). This toxicity is exerted by reduction in GSH production through the CySS/glutamate exchanger (Tan et al., 1998; Zaulyanov et al., 1999; Xu et al., 2007; Grohm et al., 2010; Chen et al., 2011; Tobaben et al., 2011; Poteet et al., 2012; **Figure 4**). Mitochondrial oxidative stress and dysfunction are important preceding events promoting glutamate induced cell death in HT-22 (Suh et al., 2007; Gliyazova et al., 2013; Shah et al., 2014). Treatment with glutamate (i) alters MMP (ii) induces mitochondrial cytochrome c release (iii) release of mitochondrial AIF, which catalyzes DNA fragmentation and apoptosis. Oxidative stress activates the c-jun N-terminal kinase (JNK) and p38 mitogen activated kinase (Yang et al., 2012) pathways via apoptosis signal regulating kinase-1 (ASK1), leading to apoptosis.

The exact mechanism of glutamate induced excitotoxicity is not clear, and some believe that this process may be mediated through the activation of MAPKs and inhibition of the PI3K/Akt pathway.

*In vitro* evidence support that glutamate treated HT22 cells, exhibit a delayed and persistent activation of ERKs which contributes to oxidative toxicity (Stanciu et al., 2000). In HT-22 cell line glutamate significantly up regulates the phosphorylation of ERK1/2 while decreasing Erk3 (Gliyazova et al., 2013). Furthermore glutamate treated HT22 increase intracellular Ca^2+^ levels by means of activation of cobalt-sensitive channels (Finkbeiner and Greenberg, 1996). It is noteworthy that cell death in HT-22 is characterized as necroptosis (Nikoletopoulou et al., 2013). Moreover, increasing evidence suggests that glutamate treated HT22 cells lack caspase activation and glutamate induced cell death proceeds independently of the bcl-2 family proteins so in this cell line glutamate induced apoptosis is mediated via the caspase independent pathway which involves calpain and AIF and is accompanied by DNA ladder formation but not chromatin condensation (Zhang and Bhavnani, 2006). AIF translocation from mitochondria to the nucleus has been identified as the final step of caspase independent mitochondrial death signaling in neurons (Kinney and Slater, 1993; Rahman and Neuman, 1996; Holohean et al., 1999; Culmsee et al., 2005; Landshamer et al., 2008; Poteet et al., 2012).

### Human Teratocarcinoma Cell Line – Ntera /D1 (NT-2)

Ntera-2 cell line is derived from a pluripotent embryonal testicular carcinoma. Upon treatment with RA, NT2 precursors are differentiated into NT2N cells, which are identical to neurons, and the majority of them express iCluRs (Perovic et al., 1996; Yoshioka et al., 1997; Paquet-Durand and Bicker, 2004; Paquet-Durand et al., 2006; Podrygajlo et al., 2009). NT2 cells are immunoreactive to the cholinergic markers choline acetyl-transferase, vesicular acetylcholine transporter, and the non-phosphorylated form of neurofilament H, all indicative of motor neurons. The NT2 system may thus be well-suited for research related to motor neuron diseases (Podrygajlo et al., 2009). Additionally NT-2N cells are able to secrete amyloid precursor protein constituting a model cell line for Alzheimer’s disease studies (Di et al., 2010).

Both NMDA and non-NMDA glutamate receptor channels have been identified electrophysiologically and mRNAs for several subtypes of glutamate receptors have been detected (Younkin et al., 1993).NT-2N cells are susceptible to both NMDA and non-NMDA mediated excitotoxicity (Younkin et al., 1993; Munir et al., 1995; **Figure 3**). High concentrations of glutamate on NT-2N can also inhibit the CySS/glutamate antiporter (X_c_^-^), diminish GSH, thus causing oxidative glutamate toxicity (Munir et al., 1995; Sandhu et al., 2002; **Figure 4**).

According to Munir et al. (1995), these cells exhibit an excitotoxic response characterized by the absence of NOS and NADPH activity, while Podrygajlo et al. (2009), support that NO is involved in the excitotoxic response of NT-2. Regularly, astrocytes are the main source of NO, however, it has been shown that NT2 neurons can produce NO and therefore increase levels of cGMP (Denninger and Marletta, 1999). In this cell line hypoxia is studied in relation to excitotoxicity and it has been shown that NMDA antagonist (MK801) can offer protection against cell death thus directly connecting hypoxia and excitotoxicity NT2 cells are mainly used to study the neuroprotective potential of glutamate antagonists (Paquet-Durand et al., 2006).

Cell death in NT-2 cell does not seem to be effected by means of caspase 3 activation alone, since addition of the NMDA antagonist MK801 results in synergistic protection (Hanko et al., 2006; **Table 2**).

### Oligodendroglial Lineage Cells

Oligodendroglial lineage cells are derived from CG-4-immortalized rat O-2A progenitor cells and are established as an *in vitro* model for oligodendroglial cell studies. Failure of MK-801, to attenuate kainate-induced cell damage under excitotoxic conditions has led to the conclusion that OLCs do not express NMDARs (Yoshioka et al., 1995) and that excitotoxicity in oligodendrocytes is mediated through non-NMDA glutamatergic receptors (Yoshioka et al., 1995, 2000; Yamaya et al., 2002; Itoh et al., 2003). *In vitro* evidence support that although both CG-4 and non-immortalized rat OLCs transcribe the NMDA GluR subunit genes NMDAR1 and NMDAR2D they do not translate NMDAR1 GluR protein (Peterson and Moore, 1980).

Excitotoxic stimuli can damage oligodendroglial cells by means of Ca^2+^ entry which leads to Ca^2+^ dyshomeostasis and mitochondrial membrane alterations. These changes can lead to release of cytochrome c and AIF (Susin et al., 1999) exhibiting a proapoptotic action. Death of oligodentroglial cells can be either caspase-depended or caspase- independent (Alberdi et al., 2002; Sanchez-Gomez et al., 2003; Ness et al., 2004). The molecular events observed after glutamate excitotoxicity are shown in **Figure 3**. Excitotoxic stimuli results in increased production of ROS, depolarization of the mitochondrial membrane and release of caspase-activating proapoptotic factors. Whether cell demise is mediated via necrosis or apoptosis, it is determined through the relative contribution of the above events (Ankarcrona et al., 1995; Sanchez-Gomez et al., 2003). Consequently, cells die via apoptosis or necrosis, as is observed in excitotoxic models (Bonfoco et al., 1995). Overexpression of Bcl2 and Bcl-xl can prevent cell death in cases of mild excitotoxic insults, whereas other members of this family such as Bad or Bax have a proapoptotic role and are involved in excitotoxicity induced mitochondrial dysfunction (Xiang et al., 1998; **Table 2**).

### C6 Cell Line

Although it has been shown that C6 rat glioma cells express iGluRs (Singh and Kaur, 2009; Tsuchioka et al., 2011; Veenman et al., 2012), it seems that excitotoxicity in this cell line is mediated by mGluRs (Albasanz et al., 1997; Viwatpinyo and Chongthammakun, 2009; Castillo et al., 2010a,b; Piers et al., 2012; Sun et al., 2012). Concurrently studied C6 and PRCNs cells, showed that in PCRNs cells mGluRs levels are down regulated and the death rate is increased, after treatment with glutamate, compared to C6 cells which were resistant to excitotoxicity, and after 24/48 h the levels of mGluRs were increased. GSH depletion seems to be responsible for the development of oxidative stress in C6 cells (Han et al., 1997; Sun et al., 2012; **Figure 4**).

Zhu et al. (2012) showed, that oxidized extracellular Cys/CySS (CySS) redox state in C6 glial cells, induced a significant increase in mGluR5-mediated phosphorylation of ERK kinases. Cys/CySS redox could be blocked by U0126 an inhibitor of MEK/MAPK and a specific mGluR5 antagonist, 2-methyl-6-(phenylethynyl) pyridine (MPEP). Activation of mGluR5 by oxidized extracellular Cys/CySS affected the expression of NF-κB and inducible iNOS (Zhu et al., 2012).

Moreover A1-mediated adenylyl cyclase inhibition and A2A-mediated adenylyl cyclase stimulation were, respectively, increased and decreased after glutamate exposure (Castillo et al., 2010b).

Neuroprotective responses activated due to loss of neural network and cell death were, the increased expression of the stress protein HSP70 (Du et al., 2012), chaperone GRP78 (Suyama et al., 2011), and of BDNF (Viwatpinyo and Chongthammakun, 2009; Hirata et al., 2012).

### Primary Rat Astrocytes

Primary rat astrocytes are widely used to investigate the protective effect of glial cells against excitotoxic neuronal trauma, specifically via astrocytic glutamate transporters GLT-1 (EAAT2) and GLAST (EAAT1), which are the main glutamate transporters in the CNS, taking up most of excess glutamate from the synaptic cleft to prevent excitotoxic neuronal death (Lu et al., 2008; Fang et al., 2012; Lane and Lawen, 2013; Foran et al., 2014; Karki et al., 2014; Obara-Michlewska et al., 2014; Sulkowski et al., 2014). Although astrocytes appear to be capable of expressing all five of the known EAAT isoforms (i.e., termed EAATs 1–5) in humans, the rodent glutamate aspartate transporter GLAST; ortholog of human EAAT1, and the glutamate transporter 1 GLT-1; ortholog of human EAAT2, are thought to be the predominantly expressed glutamate transporters in rodent astrocytes (Lane and Lawen, 2013; Karki et al., 2014) in their effort to prove that raloxifene (RX) upregulates glutamate transporters in rat primary astrocytes, found that this happens through the activation of multiple signaling pathways including ERK, EGFR, and CREB mediated by estrogen receptors (ERs) ER-a, ER-b, and GPR30. At the transcriptional level, NF-κB played a critical role in RX-induced GLT-1 expression, while TNF-a reduced GLT-1 promoter activity, mRNA and protein levels in primary astrocytes.

Ionotropic glutamate receptor or mGluRs are known to be expressed in cultured astrocytes. Although kainate receptor activation was not directly assessed, clear evidence for a functional expression of these iGluRs in astrocytes is lacking. Groups II and III mGluRs do not appear to be expressed by rodent astrocytes under standard culture conditions (Lane and Lawen, 2013).

Furthermore, potassium channels Kir4.1 abundantly expressed in astrocytes contribute to K^+^ spatial buffering, a fundamental mechanism in maintaining neuronal excitability and synaptic transmission and are implicated in the regulation of cell volume. Down regulation of Kir4.1 channels expression has been reported to decrease glutamate (Glu) uptake in cultured astrocytes. (Obara-Michlewska et al., 2014) have shown that over-activation of astroglial NMDA receptors, is a primary cause of the reduction of Kir4.1 expression in CNS disorders associated with increased exposure to Glu, giving a new insight to the excitotoxic contribution of NMDARs.

### Primary Cortical Rat Neurons

Despite of all the disadvantages pertaining to their culture, PRCNs are widely used for excitotoxicity experiments, because they present all the characteristics of living neurons activated by iGluRs agonists (Chen et al., 2013). Additionally the activation of NMDARs was verified after incubation with NMDA (Antonelli et al., 2004; Smialowska et al., 2012; Voulgari-Kokota et al., 2012; Yang et al., 2012).

Kim et al. (2012) in an effort to prove the anti-inflammatory effect of *Vitis amurensis* found out that excitotoxicity was induced not only by NMDARs but also due to the depletion of GSH levels and lipid peroxidation. MAPKs, cyclooxygenase-2 (COX-2), and pro-apoptotic proteins were also found to be active in these neurons.

Yang et al. (2012) supported the involvement of MAPK pathways in NMDAR-induced apoptosis of rat cortical neurons. Current studies show that treatment of cortical neurons with glutamate resulted in an increase in Bax with a decrease in Bcl-2 proteins, loss of the MMP followed by a release of cytochrome c, and activation of caspase-9, representing the classical mitochondrial apoptotic pathway (**Figure 3**).

Concerning neuroprotection, adenosine A2A receptor subtype stimulation induced the activation of Akt-GSK-3b signaling pathway. The blockade of this signaling pathway with specific inhibitors abolished the increase of BDNF production, possibly via modulation of ERK1/2- CREB pathway. The physiological roles of A2A receptor-induced BDNF production was demonstrated by the protection of neurons from the excitotoxicity and increased neurite extension, as well as synapse formation from immature and mature neurons. Taken together, activation of A2A receptor regulates BDNF production in rat cortical neurons, which provides a neuroprotective action (Jeon et al., 2011; **Table 1**).

### RGC-5 (Retinal Ganglion Cells)

Retinal ganglion cell (RGC-5) line is used widely in glaucoma research. This cell line simulates retinal ganglion cells as it is designated positive for certain characteristics of retinal ganglia, including Thy-1 and Brn-3C expression, and for sensitivity to glutamate excitotoxicity upon neurotrophin withdrawal. RGC-5 cell line has been widely used to study the X_c_^-^ antiporter because retina is extremely vulnerable to oxidative stress (**Figure 4**). It must be noted that there is controversy concerning the origin of the RGC-5 cell line. Van Bergen et al. (2009) have shown utilizing mitochondrial and nuclear DNA analysis that the cell line is of mouse origin (*Mus musculus*) and not of rat origin as originally was thought. Furthermore, recent evidence has surfaced from Krishnamoorthy et al. (2013) which point out that the RGC-5 cell line may have been mischaracterized and actually be is cell line 661W, a mouse SV-40 T antigen transformed photoreceptor cell line. Thus, caution is advised in drawing conclusions from data extrapolated from experiments using RGC-5 cell line as well as using the aforementioned cell line as a retinal ganglion cell line.

Retinal ganglion cells express σ1-receptor, which is believed to induce neuroprotection against excitotoxicity. Hayashi and Su have shown that σ1 receptors are localized both in the ER and on the plasma membrane in many organs including the eye. The receptor is mainly localized in ER, with two transmembrane regions that have the ability to translocate to the plasma membrane upon agonist stimulation or under stressful conditions. This translocation probably gives σ1 receptor the ability to reach plasma membrane and regulate membrane channels including voltage-gated and ligand-gated Ca^2+^, K^+^, Na^+^, Cl^-^, and SK ion channels (Hayashi and Su, 2003; Tchedre and Yorio, 2008). *In vitro* exposure of cultured rat brain neurons to selective σ-receptor ligands protects cells against glutamate or NMDA exposure (Lesage et al., 1995). Both excitotoxicity and oxidative glutamate toxicity have been proposed as possible mechanisms of RGC injury and cell death (Almasieh et al., 2012).

Moreover, *in vitro* glutamate treatment increased BDNF mRNA and protein expression and also caused a release of BDNF in the culture media (Jeon et al., 2011). NF-κB activation was observed in response to glutamate treatment and it is postulated that increased BDNF expression is stimulated through NF-κB activation (Fan et al., 2006). Also CaMKII inhibitor, AIP, may play a neuroprotective role by enhancing the release of BDNF in glutamate treated RGC-5 cells. The BDNF/TrkB signaling pathway plays a pivotal role in RGC survival (Dahlmann-Noor et al., 2010; Almasieh et al., 2012). Decline in the BDNF/TrkB signaling is an important observation in the RGCs in glaucoma disease (Gupta et al., 2013). Over activation of glutamate receptors and the resulting Ca^2+^ overload stimulates calpains which target cytoskeletal proteins, kinases and phosphatases, membrane receptors and transporters (Xu et al., 2009; **Figure 3**). Concerning the TrkB receptors, it is well-established that the trkB gene encodes a full length receptor tyrosine kinase (TrkB.FL; Tsuchioka et al., 2011). Under excitotoxic conditions calpain mediates TrkB.FL cleavage results in up regulation of the truncated TrkB isoform (TrkB.T). This is pivotal for cell survival since it results in the inactivation of RhoA-GTPase and downstream inhibition of pro-death pathways of p38/MAPK and JNK/c-Jun signaling. BDNF stimulation of TrkB receptors results in the activation of intracellular signaling pathways of Akt and the MAPKs extracellular signal-regulated kinases 1 and 2 (ERK 1/2), thus promoting cell survival (Klocker et al., 2000; Cheng et al., 2002).

### SCN2.2 Cell Line

The hypothalamic SCN is a brain region that controls circadian rhythms and is endogenously resistant to excitotoxicity *in vivo* (Ebling, 1996). This *in vivo* resistance was first established in the 1980s (Peterson et al., 1989; Reynolds and Hastings, 1995). *In vitro* studies of the ability of these cells to resist the toxic effects of glutamate would probably be the first step for excitotoxicity treatment.

It has been demonstrated that SCN2.2 (immortalized SCN cell line derived from rat SCN) retains resistance to glutamate toxicity under conditions that are toxic to other immortalized cell lines. This ability may provide insights into signaling pathways that offer endogenous neuroprotection in SCN. SCN2.2 cells retain the ability to respond to glutamate since *in vitro* studies have proved the presence of functional NMDAR subunits NR2A, NR2B, and NR2D. This fact enforces the perception that lack of excitotoxicity in SCN2.2 is not because of incapability to respond to the glutamate stimuli (Bottum et al., 2010). Bottum et al. (2010), supported that excitotoxic resistance in SCN2.2 is an endogenous ability and is not due to glutamate uptake by glia. In addition, experimental evidence support that SCN2.2 have higher levels of AkT activity than other cell lines (Marchetti et al., 2004). Activation of Akt pathway (PKB/Akt/PI_3_) is correlated with neuroprotection since Akt pathway stimulates the expression of many neuroprotective factors such as: estrogens, brain-derived neurotrophic factor (Jeon et al., 2011), insulin-like growth factor and tumor necrosis growth factor (Marchetti et al., 2004; Johnson-Farley et al., 2007; De et al., 2008; Bourque et al., 2009).

*In vitro*, SCN2.2 excitotoxic resistance was evaluated in comparison with GT1-7 (a hypothalamic cell line derived from embryonic GnRH) which served as a neuronal control and has been widely used in neurotoxicity studies (Mellon et al., 1990; Bonfoco et al., 1996; Karmarkar et al., 2011). Glutamate treated SCN2.2 and GT1-7 (10 mmol/L) resulted in activation of different signaling pathways. SCN2.2 exposure to glutamate resulted in activation of the anti-apoptotic ERK/MAPK pathway without affecting the pro-apoptotic p38/MAPK pathway, whereas in GT1-7 was noted an increase in p38/MAPK pathway and decrease in ERK/MAPK pathway (Karmarkar et al., 2011; **Table 1**).

Moreover in GT1-7 cells glutamate treatment resulted in increased levels of caspase-3 and BID protein, with subsequent DNA damage and cell death, while in SCN2.2 glutamate didn’t affect caspase-3 activity. Increased caspase 3 activity and cell death in SCN2.2 was noticed only after pretreatment with PD98059 (inhibitor of ERK/MAPK) which resulted in NMDAR-mediated death via an apoptotic pathway (Karmarkar et al., 2011).These results support the connection between activation of ERK/MAPK pathway and cell survival (Villalba et al., 1997; Persons et al., 1999; Yazlovitskaya et al., 1999). More specifically, activation of ERK/MAPK pathway stimulates the release of molecules such as (a) CREB (Cao et al., 2008) and (b) mammalian target of rapamycin (Hay and Sonenberg, 2004). The activation of the above intracellular molecules through ERK/MAPK pathway plays a key role to cell survival. It is worth mentioning that treatment with BDNF also up regulates ERK/MAPK pathway (Hetman et al., 1999; Han and Holtzman, 2000; Hetman and Kharebava, 2006).

## Conclusion

From the information presented above becomes evident that excitotoxicity is a multifactorial and complex phenomenon. Depending on the extracellular glutamate concentration (below 20 μM) the glutamate receptors alone come into play increasing the intracellular Ca^2+^ concentration. In higher extracellular glutamate concentrations, besides the action of glutamate receptors, the antiporter X_C_^-^ exchanges intracellular CySS for glutamate, thus eventually depleting the cells of their GSH reducing potential. In addition in certain conditions the reverse action of glutamate transporters (EAATs) contribute to increased extracellular glutamate concentration.

Different cell lines differ in their responses when exposed to glutamate. In some cell lines glutamate toxicity is exerted through over activation of NMDA, AMPA, or kainate receptors whereas in other cell lines lacking such receptors, the toxicity is due to glutamate induced oxidative stress (**Table 2**). Another point worth mentioning is the differentiation of temporal characteristics of the signaling cascades in different cell lines, such as time and extent of activation, that play a pivotal role in promoting either cell survival or cell death. Different researchers used different exposure times and varying concentrations of glutamate to study excitotoxicity in their cell model systems.

The evolving understanding of cell death mechanisms also contributed in part to the hazy picture of excitotoxic cell death. Today, we understand many types of cell death, necrosis and programmed cell death. Programmed cell death can be caspase dependent (apoptosis), caspase independent calpain dependent (necroptosis or apoptosis like programmed cell death) and caspase independent cathepsin dependent (autophagic cell death; Nikoletopoulou et al., 2013). The study of excitotoxicity should take into consideration these facts. We recognized the need of a well-defined system that can distinguish excitotoxicity from oxidative glutamate toxicity and take into account our current view of necrosis or programmed cell death in order to decipher the response of the nerve cell to increased extracellular glutamate concentration.

The response of the cells under such conditions can be addressed by a concerted proteomics approach in order to assess the end result of the activation of the multiple intracellular signaling cascades initiated by the excitotoxic insult. It is also of great interest to examine the kinetics of the key pathways involved in order to assess their implication in cell survival or cell death under the stimulus of glutamate toxicity.

Herein are collected and presented the cell models, used to study excitotoxicity and the findings reported so far with respect to the defense and cell death mechanisms, elicited by elevated extracellular glutamate concentration.

## Author Contributions

AK contributed to the conception of the theme-axis of this review and to the interpretation of the collected information. ES contributed to the conception of the work and also to analysis and interpretation of the information TV contributed also to the acquisition and analysis. KP contributed to the collection and analysis of the data. All the aforementioned authors cooperated for the drafting of the review, its final approval, and agreed to be accountable for all aspects of the work in ensuring that questions related to the accuracy or integrity of any part of the work are appropriately investigated and resolved.

## Conflict of Interest Statement

The authors declare that the research was conducted in the absence of any commercial or financial relationships that could be construed as a potential conflict of interest.

## Abbreviations

AIF: apoptosis inducing factor
AMPAR: α-amino-3-hydroxy-5-methyl-4-isoxazolepropionic acid receptor
BDNF: brain derived neuron factor
BID: BH3 interacting domain death agonist
cAMP: cyclic adenosine monophosphate
CNS: central nervous system
CREB: cAMP response element binding protein
Cys: cysteine
CySS: cystine
EAAT: excitatory amino-acid transporter
ER: endoplasmic reticulum
GAPDH: glyceraldehyde 3-phosphate dehydrogenase
GSH: glutathione
iGluR: ionotropic glutamate receptor
KARs: kainic acid receptors
MAPK: mitogen-activated protein kinase
mGluRs: metabotropic glutamate receptors
NADPH: nicotinamide adenine dinucleotide phosphate
NGF: nerve growth factor
NMDAR: *N*-methyl-D-aspartate receptor
NOS: nitric oxide synthase
PKC: protein kinase C
PLC: phospholipase C
PRCNs: primary rat cortical neurons
RA: retinoic acid
Src: proto-oncogene tyrosine-protein kinase
VDCC: voltage-dependent calcium channel
X_C_^-^: glutamate/cystine antiporter.
